# Computational ethology: a new path for studying canine behavior genomics and human mental disorder

**DOI:** 10.1093/nsr/nwaf558

**Published:** 2025-12-08

**Authors:** Xinyu Huang, Pengfei Wei, Guo-Dong Wang

**Affiliations:** State Key Laboratory of Genetic Evolution & Animal Models, Kunming Institute of Zoology, Chinese Academy of Sciences, China; Kunming College of Life Science, University of Chinese Academy of Sciences, China; CAS Key Laboratory of Brain Connectome and Manipulation, Shenzhen-Hong Kong Institute of Brain Science, Shenzhen Institute of Advanced Technology, Chinese Academy of Sciences, China; Guangdong Provincial Key Laboratory of Brain Connectome and Behavior, the Brain Cognition and Brain Disease Institute, Shenzhen Institute of Advanced Technology, Chinese Academy of Sciences, China; State Key Laboratory of Genetic Evolution & Animal Models, Kunming Institute of Zoology, Chinese Academy of Sciences, China; Kunming College of Life Science, University of Chinese Academy of Sciences, China; Yunnan Key Laboratory of Molecular Biology of Domestic Animals, China

Given the long-standing co-evolutionary relationship between humans and dogs, it is crucial to adopt appropriate methodologies for systematically quantifying the behavior of domestic dogs to understand the mechanisms that underlie the unique emotional bonds between species. Interest in canine behavior dates back to the renowned Charles Darwin and Ivan Pavlov, but systematic, large-scale behavioral research began to flourish in the late 1990s. Feuerbacher and Wynne systematically reviewed the development of modern canine behavioral research, with explicit discussion of its origins in the late 1990s, when several scientific papers were independently published by research groups worldwide [1]. Subsequently, an increasing number of scientific research efforts were devoted to this field, leading to significant advances.

***Current status of canine behavioral research***. Dogs present unique advantages for behavioral measurements. Their long companionship with humans has resulted in shared cognitive characteristics, particularly in terms of social cognition, emotion and behavior [2,3]. In addition, their large and diverse populations produced by artificial selection provide a wide range of behavioral variations. Compared with other animal models, dogs are distinct because of their sociability, accessibility, trainability and high similarity to humans (e.g. shared genetics, overlapping neurophysiology, and convergent social cognition), thereby making them particularly suitable for behavioral research.

Modern canine behavioral research began with the development of behavior measurements. A well-established and reliable approach is the behavior test, which assesses observable actions in controlled settings. Another one is the cognitive test focusing on specific targets and mental processes such as memory, learning or problem-solving. In the late 1990s to early 2000s, a common methodological approach involved professionals observing an individual dog’s behavior and rating it on Likert-type attitude scales [4]. Although observer-rated scales provided valuable knowledge, they were limited by inter-rater variability and reliance on experienced evaluators. To address these challenges, later studies increasingly adopted more objective measures, for instance binary outcomes (e.g. correct vs. incorrect responses in gesture comprehension tasks, which assess the capacity to interpret social cues with human beings) and total time (e.g. time spent in the unsolvable task, which evaluates the ability to seek human help) [5].

An alternative method for quantifying behavior involves utilizing questionnaire surveys. Behavioral data are collected by administering predefined questionnaires to evaluate various aspects of behavior, e.g. the Canine Behavioral Assessment & Research Questionnaire (C-BARQ) [6]. The advent of citizen science and the internet has led to its marked increase in recent years.

***Computational ethology: the path to the future***. One drawback of the methods mentioned above is that despite attempting to record behavior accurately, details are usually missing due to the limitations of human observation and the relatively low-dimensional nature of the research methods employed. Inaccuracies due to subjective evaluations and inevitable disagreements among different owners and experimenters can significantly impact the reliability and validity of behavioral assessments and research conclusions. Two independent studies yielded conflicting conclusions regarding the association between genotype and behavioral phenotype using questionnaire data [7,8]. Such inconsistencies highlight the need for more comprehensive, objective and high-dimensional approaches.

Here, we propose the concept of computational ethology as a new path to overcome these limitations by obtaining comprehensive, accurate measurements of canine behavior (Fig. 1). The most defining feature is the high-dimensional data acquisition, which entails high-resolution, automated and objective behavioral measurements that integrate hundreds of quantifiable parameters (e.g. spatial–temporal dynamics, body movements) and capture fine-grained details at millisecond precision. The other key feature is its capacity for non-invasive behavioral quantification and it can be applied to companion dogs. In comparison, computational neuroethology, a related concept, typically involves an invasive approach to record neural responses in order to investigate the neural mechanisms underlying behavior and is more suitable for model organisms under laboratory conditions [9].

**Figure 1.**
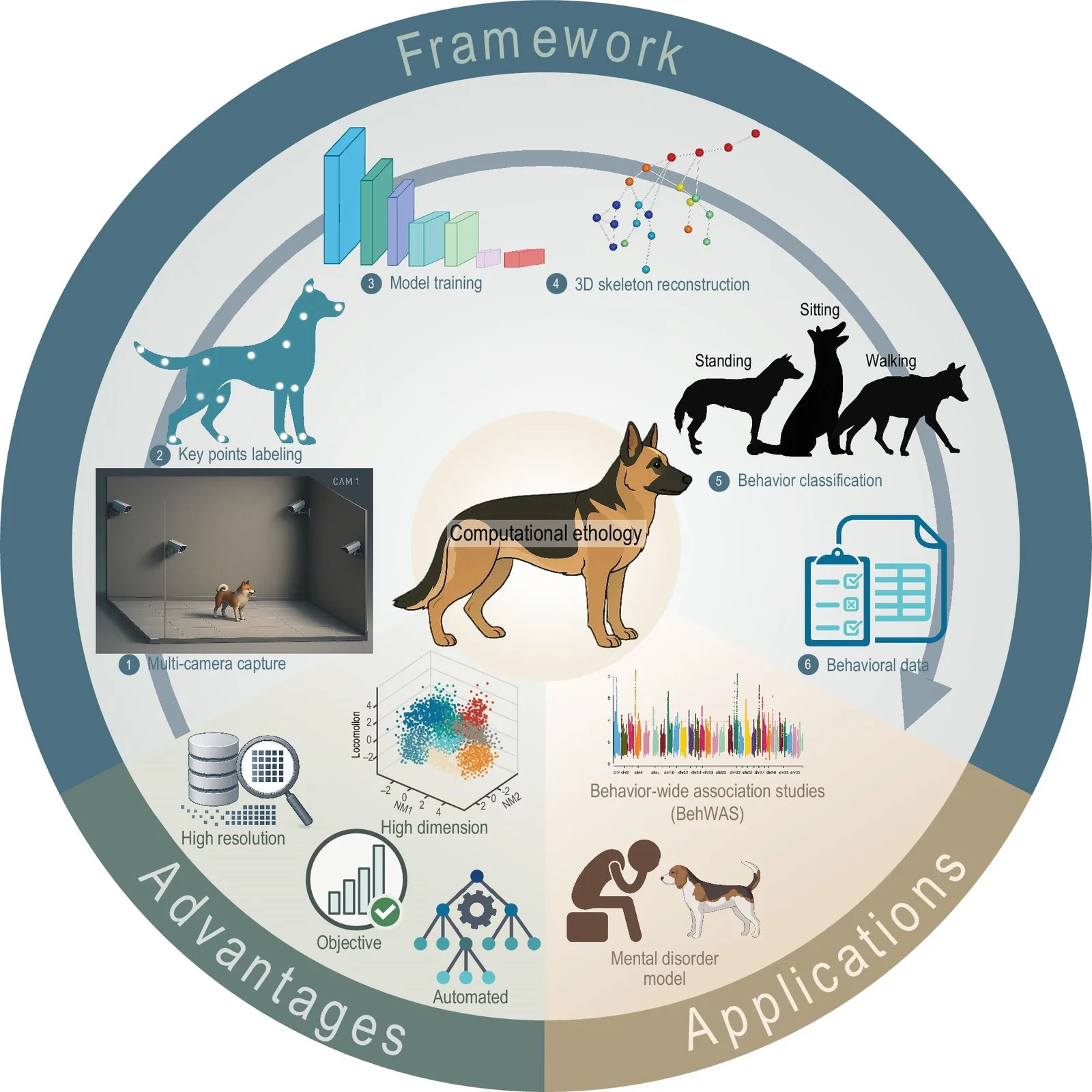
The core framework, advantages and application of computation ethology. The framework (top) details the analytical steps of computational ethology: (1) multi-camera capture; (2) labeling of key points; (3) 3D skeleton reconstruction; (4) behavior classification (e.g. sitting, standing, walking); and (5) behavioral data acquisition. This approach provides key advantages (bottom left), including high-dimensional, high-resolution, objective and automated data acquisition. These capabilities can facilitate key applications (bottom right) such as BehWASs and human mental disorder models for decoding the genetic or neural mechanisms of behavior.

Based on disciplines such as mathematics, engineering and machine learning, computational ethology has the potential to surpass the technical limitations of conventional approaches such as low dimensionality and reliance on human observations. By leveraging recent advances in machine learning and computer vision, computational ethology can automatically identify and quantify known, observer-defined behaviors, as well as discover potentially new behaviors exhibited by freely moving animals without constraint. The process begins by utilizing a set of high-resolution, calibrated cameras to simultaneously record animal behavior with an automated 3D behavior capture system. Key points on the animal are then labeled for detection, enabling tracking of the animal’s body skeleton and the acquisition of hundreds of quantifiable parameters. Subsequently, different behavioral types can be distinguished by applying artificial intelligence-driven supervised or unsupervised learning techniques (such as convolutional neural networks, transformers and mathematical frameworks, e.g. graph theory), leading to the production of a comprehensive ethogram. This technique enables the design of diverse test paradigms and records behavior at an unprecedented resolution, thereby revolutionizing behavioral study [10].

Computational ethology has been applied in several studies to explore canine behavior, yielding novel findings. For instance, Han *et al*. introduced a new framework for tracking multiple animals, including dogs, and classifying their interactions to expand the applications of computational ethology [11]. Ren *et al*. produced a framework for monitoring the tail-wagging behavior of dogs during interactions with humans, and found that each dog exhibited distinct and consistent tail-wagging characteristics, where tail-wagging tended to develop asymmetrically from the left side to the right side during dog–human interactions [12]. These studies highlight the significant potential of computational ethology as a powerful tool for advancing dog behavior research in individual behavior performance, as well as in multi-individual interactions, e.g. cooperative behavioral paradigms.

***Computational ethology facilitates canine behavior genomics*.** The emerging field of computational ethology has immense potential for revolutionizing the study of canine behavior. By enabling the comprehensive and integrated understanding of behavior, computational ethology can significantly advance our knowledge in areas such as behavioral genetics. Given that behaviors are complex traits influenced by genetics, environment and immediate context, traditional research methods are inadequate or inconsistent [6,7]. Computational ethology facilitates comprehensive behavioral assessments to allow researchers to connect behavioral phenotype with genetic data and environmental influences. In parallel with genome-wide association studies (GWASs), phenome-wide association studies (PheWASs) employ a specific genotype as their foundation to investigate its associations with a broad spectrum of phenotypes [13]. Here, we propose behaviome-wide association studies (BehWASs) to capture the connection between behavior and genetics [6,7,14]. High-dimensional behavioral data would be systematically tested against genetic variations, enabling the discovery of complex genotype–behavior relationships across the entire ethogram. Methodological challenges include the multiple-hypothesis testing problem inherent in analyzing high-dimensional behavioral data and the valid distillation of biologically meaningful behavioral phenotypes from hundreds of quantifiable behavioral parameters. Taken together with genome-wide selective scans, it can exploit the growing wealth of canine genetic and high-dimensional behavioral data and has great potential for uncovering the complex interplay between genes and behavior in dogs.

***Dog model as an ideal model for studying human mental disorders***. As a traditional yet promising model, the dog model also plays a crucial role in studying human mental disorders. They have undergone a remarkable co-evolution history [3] and have behavioral parallels with humans [15]. Sophisticated social-cognitive skills and emotional bonding with human beings enable scientists to investigate behavioral paradigms similar to humans but not feasible in other biomedical models. This is where computational ethology emerges as an innovative tool, offering a comprehensive view of behavioral characteristics. Computational ethology can not only draw inspiration from traditional experimental designs used in rodent and non-human primate studies to create innovative behavioral paradigms, but also shed new light on the analysis of mental disorders, offering fresh insights into their underlying mechanisms, which maximizes the potential of the dog as an ideal model for studying human behaviors.

It’s crucial to establish robust ethical frameworks when utilizing the dog as a model for human health. For studying naturally occurring behaviors or disorders in companion dogs, the primary goal should be the direct improvement of canine welfare by prioritizing the dogs’ emotional well-being and benefiting the dogs involved. Strict privacy safeguards like transparent informed consent and clear data ownership are also vital for pet owners. For using laboratory beagle dogs as a biomedical model, it’s essential to adhere to a range of ethical and welfare standards of model animals, among which the key requirement is the adoption of minimally invasive methods. Given the diverse ethical priorities in various research contexts, establishing a proper ethical standard remains an open question and requires more discussion.
